# Tick Saliva and Salivary Glands: What Do We Know So Far on Their Role in Arthropod Blood Feeding and Pathogen Transmission

**DOI:** 10.3389/fcimb.2021.816547

**Published:** 2022-01-19

**Authors:** Girish Neelakanta, Hameeda Sultana

**Affiliations:** Department of Biomedical and Diagnostic Sciences, College of Veterinary Medicine, University of Tennessee, Knoxville, TN, United States

**Keywords:** ticks, saliva, HSP70s, OATPs, cement, histamines, exosomes, vaccines

## Abstract

Ticks are blood-sucking arthropods that have developed myriad of strategies to get a blood meal from the vertebrate host. They first attach to the host skin, select a bite site for a blood meal, create a feeding niche at the bite site, secrete plethora of molecules in its saliva and then starts feeding. On the other side, host defenses will try to counter-attack and stop tick feeding at the bite site. In this constant battle between ticks and the host, arthropods successfully pacify the host and completes a blood meal and then replete after full engorgement. In this review, we discuss some of the known and emerging roles for arthropod components such as cement, salivary proteins, lipocalins, HSP70s, OATPs, and extracellular vesicles/exosomes in facilitating successful blood feeding from ticks. In addition, we discuss how tick-borne pathogens modulate(s) these components to infect the vertebrate host. Understanding the biology of arthropod blood feeding and molecular interactions at the tick-host interface during pathogen transmission is very important. This information would eventually lead us in the identification of candidates for the development of transmission-blocking vaccines to prevent diseases caused by medically important vector-borne pathogens.

## Introduction

Ticks are blood-feeding arthropods that transmits various bacterial, viral and protozoan pathogens to humans and animals (Azad, 2007; Wikel, 2013; Neelakanta and Sultana, 2015; Schneider et al., 2021; Sosa et al., 2021). In nature, the successful existence of ticks could be reasoned due to its ability to feed on various animals (Anderson, 1989; Richter et al., 2000; Embers et al., 2013; Linske et al., 2018; Banovic et al., 2021; Sidge et al., 2021). Ticks feed on the host by two mechanisms: pool feeding and/or piercing and sucking (Hurd, 2010; Simo et al., 2017). During pool feeding, tick biting mouthparts will cut the host skin and superficial blood capillaries/vessels that results in the leak of blood (Hurd, 2010; Simo et al., 2017). The leaked blood is later consumed by tick (Hurd, 2010; Simo et al., 2017). However, feeding by piercing and sucking type will occur when ticks cannulate their specialized mouthparts into the vertebrate host skin and blood capillaries/vessels to draw blood (Hurd, 2010; Simo et al., 2017). With either of these feeding methods, ticks can successfully acquire pathogens in a blood meal from infected vertebrate host or could also transmit pathogens to a naïve host (Wikel, 2013; Simo et al., 2017; Schneider et al., 2021)

During blood feeding, ticks secrete a plethora of components into saliva that facilitates them in acquiring a blood meal from the vertebrate host. These components include several anti-immunomodulatory, anti-vasodilatory, anti-coagulants, anti-complement factors and anti-platelet aggregation factors (Schoeler and Wikel, 2001; Brossard and Wikel, 2004; Wikel, 2013; Murfin and Fikrig, 2017; Simo et al., 2017; Nuttall, 2019). Recent research provides evidence that ticks excrete extracellular vesicles, such as exosomes (Zhou et al., 2018; Sultana and Neelakanta, 2020; Zhou et al., 2020) in its saliva to mitigate host responses during feeding. In addition, some of the studies suggest importance of tick heat shock proteins (HSPs) and organic anion transporting polypeptides (OATPs) in arthropod blood feeding and in interactions with the pathogens (Mulenga et al., 2008; Busby et al., 2012; Radulovic et al., 2014; Taank et al., 2017; Vora et al., 2017; Taank et al., 2018; Ramasamy et al., 2020). Some of the *Ixodes scapularis* proteins belonging to the salivary protein family (Salp family) are important secreted molecules in tick saliva that modulates various molecular events at tick-host interface (Das et al., 2001; Narasimhan et al., 2007; Wen et al., 2019). In this review, we will discuss the role of cement proteins, salivary family proteins, lipocalins, HSP70s, OATPs, and extracellular vesicles/exosomes in tick blood feeding and pathogen transmission. Due to the space limitation and availability of vast amount of literature, several of the other important articles related to this topic are not discussed.

## Tick Salivary Gland

The tick salivary gland is an important organ that is not only required to produce saliva but also is important for the survival of ticks during off-host period (Fawcett et al., 1981; Bowman and Sauer, 2004; Simo et al., 2017). In addition, tick-borne pathogens use tick salivary glands to colonize and replicate and use tick saliva for their transmission from vector to the vertebrate host (Simo et al., 2017). Ticks are broadly classified into hard (Ixodidae) and soft (Argasidae) ticks. The salivary glands of ticks in both groups consists of two to four types of acini (Fawcett et al., 1981; Bowman and Sauer, 2004; Simo et al., 2017). The salivary glands of female ticks in Ixodidae group consists of type I, II and III acini (Fawcett et al., 1981; Walker et al., 1985; Bowman and Sauer, 2004; Simo et al., 2017). Whereas type IV acini are specifically present in the salivary glands of male ticks in Ixodidae group (Feldman-Muhsam et al., 1970; Bowman and Sauer, 2004). The salivary glands of ticks in Argasidae group consists of type I and II acini (Bowman and Sauer, 2004; Simo et al., 2017). Type I acini are agranular and type II and III are granular acini (Bowman and Sauer, 2004; Simo et al., 2017). **Table 1** shows the list of common types of cells present in each acini present in the salivary glands. Briefly, type I acini are believed to participate in water balance during tick off-host phase (Fawcett et al., 1981; Bowman and Sauer, 2004; Sonenshine and Roe, 2014; Simo et al., 2017). A study from Needham and colleagues (Needham et al., 1990) has indicated that presence of lipid inclusions in type I acini was associated to produce hygroscopic saliva for maintaining water balance during tick off-host periods. Type II acini consists of six (a, b, c_1_, c_2_, c_3_ and c_4_) types of granular cells (Fawcett et al., 1981; Walker et al., 1985; Bowman and Sauer, 2004; Sonenshine and Roe, 2014; Simo et al., 2017). Type II acini cells are associated with the production of secreted glycoproteins involved in modulation of host immune responses and proteins important in cement cone formation (Fawcett et al., 1981; Walker et al., 1985; Bowman and Sauer, 2004; Sonenshine and Roe, 2014; Simo et al., 2017). Type III acini consists of three (d, e and f) types of cells important in the formation of cement cone (Jaworski et al., 1990). In general, in unfed ticks both types II and III cells have a mean diameter of about 45μM. However, in fed ticks the mean diameter for these acini is about 150 μm (Walker et al., 1985; Simo et al., 2017). This change is due to increased morphological transformation of salivary glands during feeding (Walker et al., 1985; Simo et al., 2017). The size of type IV acini was also noted to be remarkably enlarged in ticks after feeding (Feldman-Muhsam et al., 1970; Sauer et al., 1995). The secretions from type IV acini cells are believed to be involved in the transfer of spermatophore from male to female tick during the mating process (Feldman-Muhsam et al., 1970; Sauer et al., 1995). Furthermore, type II and type III acini cells are associated to be involved in the development and replication of tick-borne pathogens (Fawcett et al., 1981; Bowman and Sauer, 2004; Sonenshine and Roe, 2014; Simo et al., 2017). These studies indicate the importance of tick salivary gland cells in blood feeding, mating and interactions with pathogens.

**Table 1:** Tick salivary gland type I, II, III and IV acini structure and role.

Acini	Presence	Structure	Role	Reference
Type I	Ixodidae Argasidae	Single central lamellate cell, multiple peripheral lamellate cells, peritubular cells, one circumlumenal cell	Involved in off-host osmoregulation	(Fawcett et al., 1981; Bowman and Sauer, 2004; Sonenshine and Roe, 2014; Simo et al., 2017)
Type II	Ixodidae Argasidae	Epithelial cells, Adlumenal cell, ablumenal interstitial cells, neck cells, a, b, c_1_-c_4_ glandular cell types	Involved in on-host osmoregulation, site of synthesis of protein and lipid factors for secretion, site of pathogen development and replication, cells contain secretory granules	(Fawcett et al., 1981; Bowman and Sauer, 2004; Sonenshine and Roe, 2014; Simo et al., 2017)
Type III	Ixodidae	Similar to type II but with d, e, f, glandular cell types	Involved in on-host osmoregulation, site of synthesis of protein and lipid factors for secretion, site of pathogen development and replication, cells contain secretory granules	(Fawcett et al., 1981; Bowman and Sauer, 2004; Sonenshine and Roe, 2014; Simo et al., 2017)
Type IV	Ixodidae (males)	Similar to type II and III but with granular cell type g	copious salivation that contributes to the efficient transfer of spermatophore to female genital aperture during mating	(Walker et al., 1985; Sonenshine and Roe, 2014)

## Tick Saliva and Its Secretion

The discovery of salivary glands as organs of fluid secretion in ticks was reported several decades ago (Tatchell, 1969). Several studies have now identified that catecholamine neurotransmitter dopamine acts as an important stimulator of salivary secretion from tick salivary glands (Sauer et al., 1995; Sauer et al., 2000; Bowman and Sauer, 2004; Simo et al., 2017). The receptor for dopamine, D1, has been reported (Sauer et al., 1995; Sauer et al., 2000; Bowman and Sauer, 2004; Simo et al., 2017). The binding of dopamine to its D1 receptor stimulates adenylate cyclase thereby resulting in increased production and release of intracellular cAMP (Sauer et al., 1995; Sauer et al., 2000; Bowman and Sauer, 2004; Simo et al., 2017). The increased intracellular cAMP level is thought to enhance protein phosphorylation in tick salivary glands (Sauer et al., 1995; Sauer et al., 2000; Bowman and Sauer, 2004; Simo et al., 2017). *In vitro* studies show that exogenously added dopamine and cAMP could stimulate fluid secretion from salivary glands (Sauer et al., 1995; Sauer et al., 2000; Bowman and Sauer, 2004; Simo et al., 2017). Recent studies also provide evidences on the participation and interaction of several molecules and pathways including cytosolic phospholipase A2 (cPLA2), intracellular calcium, voltage-dependent calcium channels, arachidonic acid, cyclooxygenase pathways, prostaglandin-E2 (PGE_2_), prostaglandin-F_2_α (PGF_2_α), EP1-like receptor, phospholipase C (PLC), G-protein coupled receptors, inositol 1, 4,5-triphosphate (IP_3_), diacylglycerol (DAG), N-ethylmaleimide-sensitive factor attachment protein receptor (SNARE) complex proteins, nSec1-like protein and synaptobrevin for exocytosis of protein secretion in tick saliva (Sauer et al., 1995; Sauer et al., 2000; Karim et al., 2002; Bowman and Sauer, 2004; Karim et al., 2004a; Karim et al., 2004b; Maritz-Olivier et al., 2007; Simo et al., 2017). These studies provide a list of molecules that participates in the signaling cascades leading to the production of saliva from ticks.

## Tick Saliva Components

The basic components of tick saliva are water, ions, non-peptide molecules, tick peptides, tick proteins, host proteins and exosomes (Vora et al., 2017; Nuttall, 2019; Villar et al., 2020; Zhou et al., 2020). Tick molecules that are secreted in saliva performs various immunomodulatory activities at the vector-host interface. The molecules involved in the modulation of host defenses are categorized based on its mechanism of action in vasodilation/vasoconstriction, wound healing/angiogenesis, platelet aggregation, blood coagulation, innate immune responses, complement system and acquired immune responses. A detailed list of all tick proteins grouped in this broad classification are excellently reviewed by Simo and colleagues (Simo et al., 2017).

## Cement Proteins

The first step of tick blood feeding starts with penetration and anchoring of its hypostome to the host skin (Schoeler and Wikel, 2001; Wikel, 2013; Sonenshine and Roe, 2014; Nuttall, 2019). Ticks make a cone-like feeding cavity on the host skin and secretes cement-like substances into the pit (Schoeler and Wikel, 2001; Wikel, 2013; Sonenshine and Roe, 2014; Nuttall, 2019). Cement is basically composed of proteins and lipids that are secreted by d and e cells of types II and III acini (Sonenshine and Roe, 2014). It has been noted that ixodid ticks secretes cement molecules between 5-30 min of the insertion of mouthparts (Suppan et al., 2018). It is believed that ticks deposit different forms of cement molecules during their entire time of feeding (Suppan et al., 2018). During the initial phase, ticks secretes core cement that solidifies rapidly (Suppan et al., 2018). However, around 24 hours after the start of feeding, ticks secrete another form of cement (cortical cement) that hardens slowly (Suppan et al., 2018). Some studies have also associated deposition of another form of cement during the final stages of tick feeding (Moorhouse and Tatchell, 1966). After taking a complete blood meal ticks detach from the host. So far, no studies have provided evidence on the mechanism on how ticks detach from the host. Some of the studies suggested that ticks could secrete several protease-like molecules in its saliva to dissolve cement at the final stage of tick feeding (Bullard et al., 2016; Suppan et al., 2018). Therefore, it is reasonable to hypothesize that tick may activate proteasome pathways at the final stages of tick feeding leading cement molecules for degradation pathways. However, it remains an open question to address how ticks detach from the hardened cement cone.

Based on LC-MS/MS analysis, a recent study has reported presence of 160 *Amblyomma americanum* proteins in a cement cone (Hollmann et al., 2018). These includes 19% glycine-rich proteins, 12% protease inhibitors, 11% of proteins with unknown function, 4% mucin, 1% detoxification proteins, 1% storage proteins and 1% lipocalin (Hollmann et al., 2018). Some of the cement proteins are believed to have immunomodulatory activities (Sonenshine and Roe, 2014). Most of the ticks belonging to ixodid group secrete cement proteins during feeding (Sonenshine and Roe, 2014). However, *Ixodes holocyclus* (predominantly found in Australia) was not noted to be secreting cement proteins during feeding (Binnington and Stone, 1981). A recent proteomic analyses study has reported that *Rhipicephalus microplus* sialome and cementome consists of both host- and tick-derived proteins (Villar et al., 2020). Biomolecules such as alpha-Gal was also noted to be present in the siaolome and cementome of this tick (Villar et al., 2020). In addition, chemical elements such as C, O and N were noted to present in high abundance and S, P, Cl, Na and K were noted to be present in low abundance (Villar et al., 2020). The authors hypothesized that collectively these molecules are involved in tick cement formation, solidification, and maintenance to aid in attachment, blood feeding, modulate host responses at the bite site and detachment (Villar et al., 2020).

Several studies have provided evidence that blocking cement function could significantly affect tick-feeding efficiency (Mulenga et al., 1999; Bishop et al., 2002; Trimnell et al., 2002; Labuda et al., 2006; Zhou et al., 2006; Havlikova et al., 2009). In addition, it was noted that immunization of animals with *Haemaphysalis longicornis* cement protein, with a molecular mass 29 kDa, followed by tick feeding resulted in 40 to 56% mortality rate in larvae and nymphs (Mulenga et al., 1999). A study has demonstrated that immunization of animals with cement proteins also blocked transmission of tick-borne encephalitic virus (TBEV) from infected nymphal ticks to the murine host (Labuda et al., 2006). Collectively, these studies indicate that cement proteins are critical for ticks to complete a blood meal.

## Salivary (Salp family) Proteins

Das and colleagues screened for *Ixodes scapularis* salivary antigens that elicit antibodies in the host (Das et al., 2001). They screened 100,000 *I. scapularis* library clones with sera obtained from rabbits infested with ticks (Das et al., 2001). The antibody-screening assay identified 47 clones that encoded 14 different *I. scapularis* genes (Das et al., 2001). Based on the molecular mass the authors designed these clones as Salp9, Salp10, Salp13, Salp14, Salp15, Salp16A, Salp17, Salp25A, Salp25B, Salp25C, Salp25D, Salp26A and Salp26B (Das et al., 2001). Salp9 amino acid sequence was noted to have 50% similarity to anticomplement protein, ISAC, from *I. scapularis* (Das et al., 2001). A study that identified ISAC for the first time reported that this protein specifically inhibits regulators of complement activation of the alternative pathway, such as factor H (Valenzuela et al., 2000). Salp10 belongs to Kunitz/BPTI proteins (Das et al., 2001; Dai et al., 2012). Studies have reported that Kunitz/BPTI proteins regulate host blood supply and inhibit angiogenesis and wound healing during tick feeding (Islam et al., 2009; Paesen et al., 2009). Salp13 protein was noted to show weak similarity to vertebrate transforming growth factor (TGF-beta) superfamily of proteins (Das et al., 2001). Salp14 was noted to have no orthologs in other organisms (Das et al., 2001). However, BLASTp search performed during the writing of this review indicated that Salp14 orthologs or Salp14-like proteins are abundantly found in several *Ixodes* species including *I. scapularis* with a percent identity ranging from 60-94%. Based on the BLASTp search, we noted that Salp14 shows 62% identity with *I. scapularis* pheromone-processing carboxypeptidase KEX1-like protein. Salp15 orthologs are present in several tick species (Sultana et al., 2016; Wen et al., 2019). Several colleagues have worked on Salp15 that provided important information on the role of this protein in tripartite interactions involving vector, host, and a pathogen (Anguita et al., 2002; Ramamoorthi et al., 2005; Juncadella et al., 2007; Schuijt et al., 2008; Wen et al., 2019). Salp15 has been reported to have several immunomodulatory activities. It has been shown that Salp15 binds surface of CD4+ T-cells and inhibits activation of CD4+ T-cells by modulation of TCR-mediated signaling pathways (Anguita et al., 2002; Garg et al., 2006). Hovius and colleagues have reported that Salp15 binds to DC-SIGN on dendritic cells and triggers Raf-1/MEK-dependent signaling pathway affecting cytokine profiles at transcriptional and post-transcriptional levels (Hovius et al., 2008). These changes were noted to modulate Toll-like receptor-induced DC activation (Hovius et al., 2008). In addition, it was reported that binding of Salp15 to *Borrelia burgdorferi* outer surface protein C (OspC) protects these spirochetes from antibody-mediated killing and phagocytosis (Ramamoorthi et al., 2005; Schuijt et al., 2008). The expression of Salp16 was reported to be induced during tick feeding (Das et al., 2000). Further studies, including our previous study, indicated that *Anaplasma phagocytophilum* phosphorylates tick actin to selectively regulate Salp16 gene expression (Sultana et al., 2010) critical for this bacterial survival in tick salivary glands (Sukumaran et al., 2006). From the BLASTp search performed during the writing of this review we noted that Salp17 shows approximately 83% identity with *I. ricinus* lipocalin. However, future studies need to confirm if this molecule is indeed a lipocalin or not. It was reported that *A. phagocytophilum* downregulates Salp10, Salp13 and Salp17 (Sukumaran et al., 2006). Future studies need to unravel the significance of this downregulation in tick-pathogen interactions. Tyson and colleagues have reported that recombinant Salp20 inhibited alternative complement pathway by dissociating the C3 convertase (Tyson et al., 2007). In the same study authors noted that Salp20 partially protects serum sensitive *B. burgdorferi* from lysis by normal human serum (Tyson et al., 2007). The BLASTp search revealed that Salp25A, Salp25B and Salp25C shows higher percent of identity with histamine-binding proteins from several tick species. One of the potential studies showed that Salp25D is required for acquisition of *B. burgdorferi* from infected vertebrate host to ticks (Narasimhan et al., 2007). Salp25D was shown to act as an anti-oxidant at the vector-host interface providing survival advantages for spirochetes to enter into ticks (Narasimhan et al., 2007). Overall, these studies indicate that ticks secrete several multifunctional Salp family proteins that helps ticks to get a successful blood meal.

## Lipocalins

Lipocalins are small molecules that are secreted into saliva during tick blood feeding (Flower, 1996; Mans, 2005; Mans and Ribeiro, 2008; Mans et al., 2008). Several of these molecules belonging to the lipocalin family have been identified in both hard and soft ticks (Paesen et al., 1999; Beaufays et al., 2008; Mans and Ribeiro, 2008; Mans et al., 2008; Cheng et al., 2010; Hollmann et al., 2018; Neelakanta et al., 2018). With the analysis of the available transcriptomics and proteomics data and several deposited sequences in the databases, a recent study reported that 3, 689 sequences were classified as lipocalins (Ribeiro and Mans, 2020). Of which 1,050 sequences were noted to have PFAM His bind domain (Ribeiro and Mans, 2020). Lipocalins have high diversity in its amino acid sequences. The comparative sequence analyses have revealed that some lipocalins have even less than 20% similarity among themselves (Flower et al., 1993; Mans and Ribeiro, 2008; Mans et al., 2008). Several approaches including analysis of amino acid sequence, exon-intron-structure, protein structure and machine learning have been used to classify proteins as lipocalins (Ganfornina et al., 2000; Sanchez et al., 2003; Lakshmi et al., 2015; Nath and Subbiah, 2015). Even though there could be less similarity between various lipocalins, the three-dimensional structures are conserved in some of the lipocalins (Flower et al., 1993; Flower, 1996; Paesen et al., 2000; Mans and Ribeiro, 2008; Mans et al., 2008). The core lipocalins, in which the structure is highly conserved, are grouped as “kernel lipocalins” and those that have different structure are clustered as “outliers” (Flower et al., 1993; Flower, 1996; Paesen et al., 2000; Mans and Ribeiro, 2008; Mans et al., 2008). The lipocalin structure consists of single eight-stranded antiparallel beta-barrel that winds around a central axis to form a central pocket for binding small hydrophobic molecules. In addition, lipocalins also have N-terminal alpha- and C-terminal helices (Flower, 1996; Paesen et al., 2000; Mans and Ribeiro, 2008; Mans et al., 2008). Lipocalins have an ability to form oligomers that could range from dimeric state to the formation of octamers (Keen et al., 1991; Tegoni et al., 1996). Lipocalins binds to small molecules such as histamines, serotonin and prostaglandins and modulate immune responses (Flower, 1996; Mans and Ribeiro, 2008; Mans et al., 2008). Studies have indicated that tick lipocalins inhibits platelet aggregation and complement system during tick feeding (Keller et al., 1993; Nunn et al., 2005; Mans and Ribeiro, 2008). Histamine and serotonin are derivatives of amino acids histidine and tryptophan, respectively. Upon injury, histamine is secreted by activated mast cells and basophils that allows vascular permeability and recruitment of monocytes and neutrophils to the site (Jutel et al., 2005). Platelets and mast cells secretes serotonin to induce platelet aggregation and increases vascular permeability (Van Nueten et al., 1985). The release of both these molecules from host immune cells would lead to pain and itching at the injury site resulting in the host grooming activity. To countermeasure these host responses, ticks secrete several lipocalins to bind histamines and serotonin for controlling the host grooming activities and thereby successfully attach to the host and get a complete blood meal (Keller et al., 1993; Mans, 2005; Nunn et al., 2005; Mans and Ribeiro, 2008; Mans et al., 2008).

## Heat Shock Protein 70 (HSP70)

Heat shock proteins (HSPs) are chaperones that are present in both prokaryotes and eukaryotes (Lindquist and Craig, 1988; Mayer, 2010; Piri et al., 2016; Moran Luengo et al., 2019). HSP expression is induced when organisms or cells experience hyperthermia, hypothermia, unavailability of oxygen, scarcity of ATP, free radicals, desiccation, bacterial/viral infections, presence of steroid hormones, and ethanol (Lindquist and Craig, 1988; Mayer, 2010; Piri et al., 2016; Moran Luengo et al., 2019). Among all the HSPs, HSP70 is a widely studied molecule (Lindquist and Craig, 1988; Mayer, 2010; Piri et al., 2016; Moran Luengo et al., 2019). HSP70 performs several functions in the cell (Lindquist and Craig, 1988; Mayer, 2010; Piri et al., 2016; Moran Luengo et al., 2019). This molecule acts like a quality control protein that helps in modulating misfolded proteins for re-folding or send proteins for degradation (Lindquist and Craig, 1988; Luders et al., 2000; Mayer, 2010; Moran Luengo et al., 2019). In addition, along with other co-chaperones HSP70 transports proteins into organelles (Lindquist and Craig, 1988; Luders et al., 2000; Mayer, 2010; Moran Luengo et al., 2019). HSP70 recognizes damaged proteins and along with CHIP, BAG-1 and HSJ1 ubiquitinates proteins and send them for degradation *via* proteasome (Luders et al., 2000). HSP70s have N-terminal ATPase domain followed by substrate-binding domain and a C-terminal domain. N-terminal domain is required for the hydrolysis of ATP to ADP that is critical for substrate recognition (Lindquist and Craig, 1988; Mayer, 2010; Piri et al., 2016; Moran Luengo et al., 2019). HSP70s in ATP-bound form shows high association and dissociation rates for substrate exchange. However, HSP70s in ADP-bound state have low affinity for substrate exchange (Lindquist and Craig, 1988; Mayer, 2010; Piri et al., 2016; Moran Luengo et al., 2019). Traditionally, HSP70s were considered as intracellular proteins. However, work from Tytell and Hightower laboratories provided evidence that HSP70 can also be an extracellular protein (Tytell et al., 1986; Hightower and Guidon, 1989). It is also reported that HSP70s are present in substantial quantities in serum from normal human individuals indicating its presence as an extracellular protein (Pockley et al., 1998). Several lines of evidences (reviewed in (Borges et al., 2012)) shows that HSP70 acts as immunomodulator where it could interact with dendritic cells, myeloid-derived suppressor cells (MDSCs) and monocytes. HSP70 is noted to modulate antigen presenting cells leading to increase anti-inflammatory cytokine IL-10 production (Borges et al., 2012). It is also noted that HSP70 and MyD88 increases IL-10 production (Borges et al., 2012). In addition, HSP70 is shown to play a role in anticoagulation (Rigg et al., 2016), promoting activity of Factor VIII (Ishaque et al., 2007), inhibition of plasminogen activator inhibitor-1 (PAI-1) (Uchiyama et al., 2007) and degranulation and aggregation of platelets (Rigg et al., 2016). We recently published a study providing evidence that tick HSP70 is associated with fibrinogenolytic activity during blood feeding (Vora et al., 2017). We noted that inhibition of tick HSP70 by antibody-blocking or with an inhibitor resulted in reduced degradation of fibrinogen (Vora et al., 2017). In addition, we reported for the first time that ticks and tick cells secrete exosomes in their saliva and cell culture supernatants, respectively (Zhou et al., 2020). HSP70s are highly enriched in the exosomes derived from tick saliva, salivary glands and *in vitro* cells (Zhou et al., 2018; Zhou et al., 2020). HSC70, a member of HSP70 family of proteins, from *Haemaphysalis flava* was also shown to exert anticoagulation activity (He et al., 2019). A study has reported that knockdown of HSP70 expression in tick cell line results in increased Langat virus replication (Weisheit et al., 2015). Another important study, through knockdown approach, confirmed that HSP70 is involved in tick cell responses to stress such as temperature, blood-feeding and *A. phagocytophilum* infection (Busby et al., 2012). In addition, it was noted that tick HSP70 is manipulated by *A. phagocytophilum* to increase its infectivity (Busby et al., 2012). Furthermore, the expression of HSC70 was reported to be induced in adult *Rhipicephalus haemaphysaloides* ticks upon blood feeding (Wang et al., 2019). The same study also reported that knockdown of HSC70 resulted in inhibition of blood feeding, significantly decreased tick engorgement rates and increased death rates/mortality in these ticks (Wang et al., 2019). In summary, these studies suggests that tick HSP70 may not be only important to modulate host immune responses and facilitates fibrinogenolysis at the bite site but also aids in managing stress in ticks during blood feeding.

## Organic Anion Transporting Polypeptides(OATPs)

In humans, organic anion transporting polypeptides (OATPs) are a multigene family that are involved in the transport of various hormones, drugs, anions, signaling molecules, growth factors, neurotransmitters, steroids, metabolites, antivirals, antibiotics and anticancer drugs (Hagenbuch and Meier, 2004; Roth et al., 2012; Stieger and Hagenbuch, 2014). OATPs are now considered as important molecules in pharmacokinetics (Kalliokoski and Niemi, 2009). OATPs are expressed on epithelial cells in different human organs and tissues including liver, intestine, kidney, placenta, skeletal muscle, blood brain barrier and choroid plexus (Hagenbuch and Meier, 2004; Roth et al., 2012; Stieger and Hagenbuch, 2014). Based on computer-based modelling, it is noted that OATPs have N and C terminal domains that are cytosolic and contains numerous transmembrane regions with extracellular and intracellular loops (Hagenbuch and Meier, 2004; Roth et al., 2012; Stieger and Hagenbuch, 2014). Organic anion transporters (OATs) that transport smaller molecules are shown to be the transporter for tryptophan metabolite Xanthurenic acid (XA) (Uwai and Honjo, 2013). *Ixodes scapularis* ticks encodes nine OATPs that are ubiquitously expressed in various tick tissues including midguts, salivary glands, carcass, synganglion, Malpighian tubules and ovaries (Radulovic et al., 2014). The expression of some of the OATPs were noted to be induced during tick feeding (Radulovic et al., 2014). All tick OATPs have the consensus OATP motif (WxGxWWxG) (Radulovic et al., 2014; Taank et al., 2018). Mulenga et al. (2008) have reported that expression of *Amblyomma americanum* OATP (AamOatp) was noted to be 60-80 fold higher in midgut compared to the other tissues during day1-5 blood feeding and 60-80 fold higher in ovaries compared to other tissues on day 7 post feeding. Authors indicated that these changes suggest differential role for OATPs during tick blood feeding. In addition, it was noted that knockdown of AamOatp expression resulted in ticks obtaining smaller blood meal and eventually laying fewer eggs compared to the controls (Mulenga et al., 2008). A recent study showed that arthropod OATPs protects insects from toxicity due to cardenolides, a group of cardiac-active steroids (Groen et al., 2017).

Our laboratories started working on understanding the role of OATPs in tick-pathogen interactions. Our study showed that *A. phagocytophilum* specifically regulate one of the nine tick OATPs in the *I. scapularis* salivary glands for its survival (Taank et al., 2017). We noted that expression of OATPs were upregulated upon *A. phagocytophilum* infection and with the exogenous addition of XA (Taank et al., 2017). Knockdown or functional inhibition of OATPs in ticks and tick cells by RNAi approach and inhibitor treatment, respectively, affected *A. phagocytophilum* growth (Taank et al., 2017). Follow up study from our group also showed that OATPs are regulated by tick-borne Langat virus and not by *B. burgdorferi* suggesting its role in tick-obligate intracellular pathogen interactions (Taank et al., 2018). The observation of inhibition of OATPs that significantly reduced viral replication in tick cells suggests a possible anti-viral role for these molecules (Taank et al., 2018). More recent studies showed that *A. phagocytophilum* uses tick OATPs and tryptophan pathway metabolism to inhibit build-up of reactive oxygen species that is toxic to bacterial replication (Dahmani et al., 2020). In addition, we noted that *A. phagocytophilum* regulate tick OATPs through microRNA133 (Ramasamy et al., 2020). During *A. phagocytophilum* transmission from ticks to the vertebrate host, expression of OATP was noted to be upregulated and expression of miR133 was downregulated (Ramasamy et al., 2020). Microinjection of miR133 into ticks was noted to significantly reduce OATP levels that eventually affected *A. phagocytophilum* transmission from vector to the vertebrate host (Ramasamy et al., 2020). Taken together, these studies indicates that arthropod OATPs are important molecular players in supporting bacterial/viral replication, tick blood feeding and pathogen transmission from vector to the vertebrate host.

## Extracellular Vesicles/Exosomes

In general, vesicles that are secreted outside from cells are termed as extracellular vesicles (Mellman, 1996; Fevrier and Raposo, 2004; Couzin, 2005; Keller et al., 2006; Raposo and Stoorvogel, 2013). Smaller extracellular vesicles or exosomes have a size range of 30-150 nm in diameter (Mellman, 1996; Fevrier and Raposo, 2004; Couzin, 2005; Keller et al., 2006; Raposo and Stoorvogel, 2013; Zhou et al., 2018). Initially, it was hypothesized that cells secrete exosomes as garbage vesicles to dispose waste from the cells. However, based on recent reports, exosomes are now considered as vesicles to transport various molecules including proteins, lipids, DNA, RNA, microRNA, and peptides from one cell to the other (Ludwig and Giebel, 2012; Fleming et al., 2014; Gupta and Pulliam, 2014; Van Niel et al., 2018; Pegtel and Gould, 2019). Exosomes are basically from an endosomal origin with a part of late endosomal membrane budding as intraluminal vesicles (ILVs). ILV-containing endosome is referred as multivesicular bodies (MVBs). Endosomal sorting complex required for transport (ESCRT) recruit cargo material into the limiting membrane of MVBs that releases into the lumen as ILVs. MVBs fuse with the plasma membrane to release ILVs as extracellular vesicles (Mellman, 1996; Fevrier and Raposo, 2004; Couzin, 2005; Keller et al., 2006; Raposo and Stoorvogel, 2013). Numerous studies have studied importance of exosomes in host-pathogen interactions in mammalian systems (Schwab et al., 2015; Anderson et al., 2016). Many viruses, bacteria and parasites could use exosomes as a “Trojan Horse” to send messages from one cell to the other (Gould et al., 2003; Aline et al., 2004; Bhatnagar et al., 2007; Abrami et al., 2013; Alenquer and Amorim, 2015; Schwab et al., 2015; Anderson et al., 2016; Sampaio et al., 2017; Sultana and Neelakanta, 2020). Compared to more studies that used vertebrate systems to address the role of exosomes in host-pathogen interactions none of the studies were performed on tick exosomes until we reported for the first-time showing the role of arthropod exosomes in tick-Langat virus interactions (Zhou et al., 2018). We noted that majority of the extracellular vesicles/exosomes isolated from ISE6 tick cells had a size range between 30-150 nm (Zhou et al., 2018). Several of the viral components, including viral + and -ve sense RNA strands, nonstructural protein 1 (NS1) and Envelope protein (E) was present inside the exosomes derived from Langat-virus-infected tick cells (Zhou et al., 2018). In addition, it was observed that treatment of tick cells with GW4869, an inhibitor that blocks exosome release, affected viral replication (Zhou et al., 2018; Regmi et al., 2020). These findings indicate that viruses could use exosomes to disseminate from one tick cell to another. In addition to ticks, we were also able to show for the first time that mosquito C6/36 cells also secrete exosomes (Vora et al., 2018). Exosomes derived from mosquito cells contained full genome of dengue virus 2 (Vora et al., 2018). Tick- or mosquito-derived exosomes were able to transmit Langat or Dengue viruses, respectively, to human keratinocytes indicating a possible role of arthropod exosomes in the transmission of these pathogens from vector to the vertebrate host. A more recent study from our group also showed successful isolation of exosomes from tick saliva and salivary glands (Zhou et al., 2020). Exosomes isolated from tick salivary glands inhibit wound healing in human keratinocytes by downregulating C-X-C motif chemokine ligand 12 (CXCL12) and upregulating interleukin-8 (IL-8) (Zhou et al., 2020). This is an important finding that suggests that ticks secrete exosomes in their saliva to inhibit wound healing (at the bite site) thereby facilitating arthropod blood feeding. Collectively, this is an emerging area that provides novel information on the role of exosomes in tick blood feeding and pathogen transmission from vector to the vertebrate host.

## Anti-Tick Vaccine

**Figure 1:**
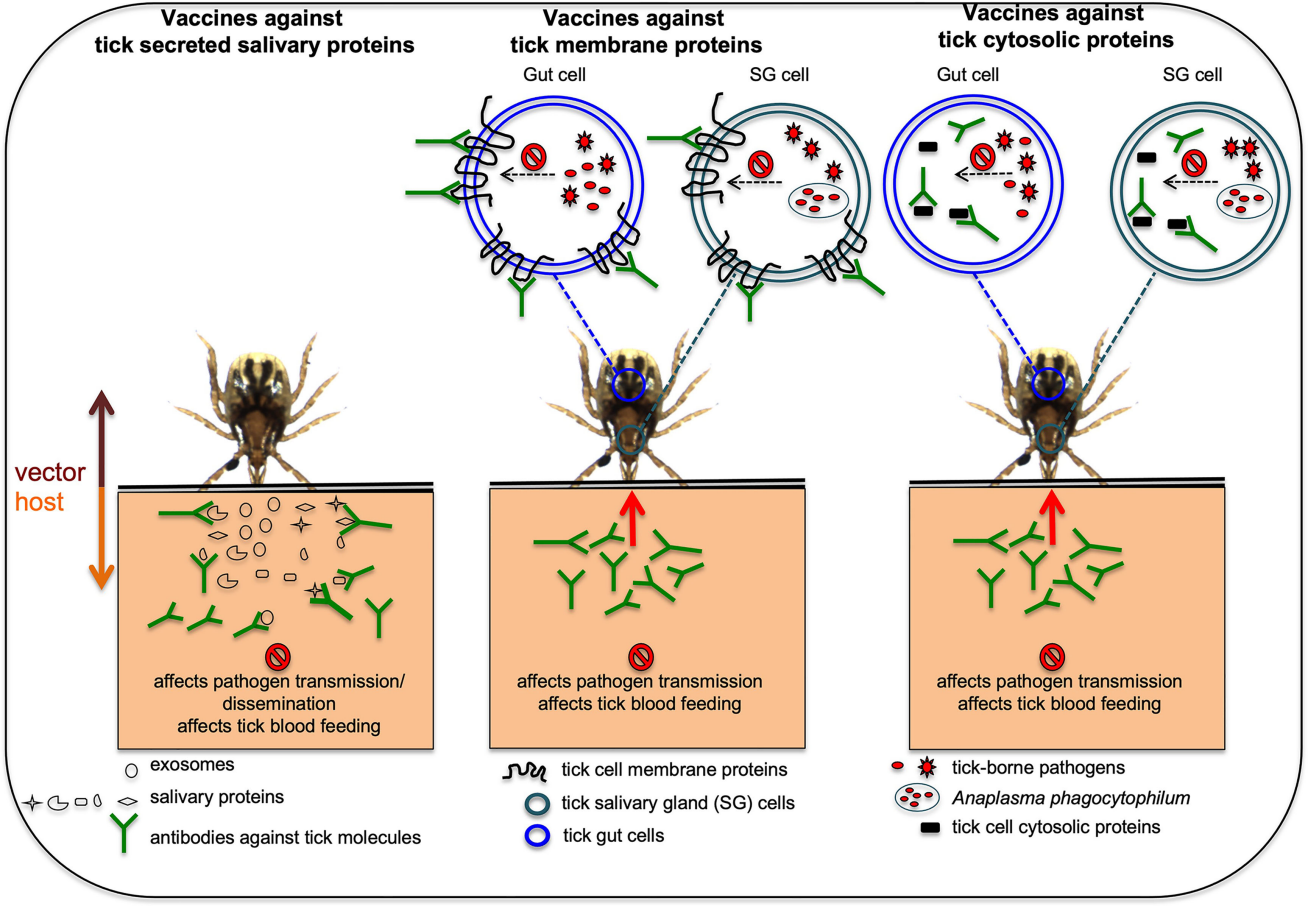
Proposed model on how anti-vector vaccines works against various tick proteins are shown. In hosts that are immunized with antibodies against tick secreted salivary or exosomal proteins that are important in blood feeding or pathogen transmission, the antibodies will bind these tick proteins and could affect pathogen transmission or dissemination and/or affect blood feeding. In hosts that are immunized with antibodies against tick membrane proteins important in blood feeding and/or pathogen transmission, the antibodies enter ticks *via* a blood meal, binds to the membrane proteins and could affect blood feeding and/or pathogen transmission. In hosts that are immunized with antibodies against tick cytosolic proteins important for blood feeding and/or pathogen transmission, antibodies enter tick body *via* a blood meal, engulfed inside cells and later binds cytosolic proteins affecting tick blood feeding and/or pathogen transmission. Picture is not drawn to the scale.

The importance of several vector molecules in tick blood feeding and in the interactions with pathogens clearly indicates that these molecules could be considered as targets for developing transmission-blocking vaccines (Merino et al., 2013; Neelakanta and Sultana, 2015). An anti-vector vaccine is a type of transmission-blocking vaccine that could be developed to target tick molecules important in pathogen acquisition, survival, and transmission (Merino et al., 2013; Neelakanta and Sultana, 2015). Several of the studies have already used animal models to test efficacy of some of tick molecules as candidates for the development of anti-vector vaccines (Willadsen, 2004; Merino et al., 2013; Neelakanta and Sultana, 2015; Valle and Guerrero, 2018; Rego et al., 2019; Tabor, 2021; Van Oosterwijk, 2021; Van Oosterwijk and Wikel, 2021). **Figure 1** illustrates how anti-vector vaccines work with different tick molecules as targets. Briefly, as ticks attaches and starts feeding, they secrete salivary molecules in saliva. In addition, ticks also secrete exosomes carrying various components including RNA, protein, peptides and miRNA (Sultana and Neelakanta, 2020; Ahmed et al., 2021; Rajendran et al., 2021). These exosomes could either fuse with the host cells and release content into the host cell or contents are released as soon as tick exosomes enters the vertebrate host. If a host is immunized with antibodies targeting tick secreted salivary or exosomal molecules (important in blood feeding or pathogen interactions) the binding of antibodies to antigens would prevent pathogen transmission from an infected tick or dissemination within the host and/or affect blood feeding (**Figure 1**). If hosts are immunized with antibodies targeting tick molecules that are transmembrane proteins (important in pathogen interactions or blood feeding eg. OATP), antibodies enter in to tick body during feeding, binds to the antigens on the membrane of the salivary gland or gut cells and affects/prevent blood feeding and/or pathogen survival/exit from these cells (**Figure 1**). Subolesin is a cytosolic and nuclear protein in ticks that participates in various signaling pathways important in blood feeding, oviposition, and interactions with pathogens (De La Fuente et al., 2011; De La Fuente et al., 2013; Shakya et al., 2014; Sultana et al., 2015). Subolesin could translocate from cytoplasm to nucleus to regulate tick gene expression (De La Fuente et al., 2011; De La Fuente et al., 2013; Shakya et al., 2014). Several studies have successfully targeted subolesin and highlighted this molecule as a strong anti-vector vaccine candidate (De La Fuente et al., 2011; De La Fuente et al., 2013; Shakya et al., 2014). Studies have reported that antibodies can enter tick cells (Munderloh et al., 1999; Blouin et al., 2003; De La Fuente et al., 2011). Therefore, it is not surprising to see the high efficacy of subolesin-based immunizations in targeting ticks. If hosts are immunized with antibodies against tick cytosolic proteins (important in pathogen interactions and/or blood feeding), antibodies enter ticks during blood feeding, engulfed into tick cells and bind to antigens in the cytoplasm. Binding of antibodies to cytoplasmic antigens could affect/prevent blood feeding and/or pathogen survival/exit from tick salivary gland or gut cells (**Figure 1**).

## Future Perspectives

With the emergence of high throughput analysis, several studies have provided enormous amount of information on proteins present in saliva and salivary glands that are important in tick feeding and/or pathogen interactions (Artigas-Jeronimo et al., 2018; De La Fuente, 2021; Martins et al., 2021; Oleaga et al., 2021). It is a daunting task to annotate several of the arthropod proteins due to lack of genome information for many tick species. However, databases like TickSialoFam (TSFam) could help in the classification of salivary proteins for further evaluations (Ribeiro and Mans, 2020). Taking this information as a foundation, future studies should start testing efficacy of anti-vector vaccines of conserved molecules not only in murine models but also in higher models including primates. The findings of exosomes in tick saliva opens several future avenues to answer some of the basic questions such as A) What is the fate of tick exosomes once it enters the vertebrate host? B) Do tick exosomes transport cargo from vector to the host cells? C) Do tick exosomes transmit pathogens from vector to the host? Some of the answers to these questions are already reported in *in vitro* cells (Sultana and Neelakanta, 2020). However, *in vivo* studies using animal models would further strengthen this emerging line of investigation. As many number of tick membrane and/or cytosolic proteins participate in vector-pathogen interactions and/or blood feeding, future studies should also focus on targeting these type of molecules. A recent study showed that anti-tick microbiome vaccines modulate tick microbiome (Mateos-Hernandez et al., 2021). Future studies could unravel whether anti-tick microbiome vaccines affect arthropod blood feeding and/or pathogen transmission. All these are interesting avenues that could further enhance in teasing out secret components of tick saliva for the development of better measures to treat and/or prevent tick-borne diseases.

## Author Contributions

GN and HS conceived, designed, wrote, read, and approved the manuscript.

## Funding

Our work in this area is supported by grants from the National Institutes of Health (NIH) R01AI130116 to GN and R01AI141790 to HS.

## Conflict of Interest

The authors declare that the research was conducted in the absence of any commercial or financial relationships that could be construed as a potential conflict of interest.

## Publisher’s Note

All claims expressed in this article are solely those of the authors and do not necessarily represent those of their affiliated organizations, or those of the publisher, the editors and the reviewers. Any product that may be evaluated in this article, or claim that may be made by its manufacturer, is not guaranteed or endorsed by the publisher.
